# Cytogenetically Elusive Sex Chromosomes in Scincoidean Lizards

**DOI:** 10.3390/ijms22168670

**Published:** 2021-08-12

**Authors:** Alexander Kostmann, Barbora Augstenová, Daniel Frynta, Lukáš Kratochvíl, Michail Rovatsos

**Affiliations:** 1 Department of Ecology, Faculty of Science, Charles University, 12844 Prague, Czech Republic; alexkostmann@gmail.com (A.K.); augstenova.barbora@gmail.com (B.A.); kratoch1@natur.cuni.cz (L.K.); 2 Department of Zoology, Faculty of Science, Charles University, 12844 Prague, Czech Republic; frynta@centrum.cz

**Keywords:** comparative genome hybridization, CGH, evolution, fluorescence in situ hybridization, FISH, heterochromatin, rDNA, karyotype, reptiles, sex determination, sex chromosomes, telomeres

## Abstract

The lizards of the species-rich clade Scincoidea including cordylids, gerrhosaurids, skinks, and xantusiids, show an almost cosmopolitan geographical distribution and a remarkable ecological and morphological divergence. However, previous studies revealed limited variability in cytogenetic traits. The sex determination mode was revealed only in a handful of gerrhosaurid, skink, and xantusiid species, which demonstrated either ZZ/ZW or XX/XY sex chromosomes. In this study, we explored the karyotypes of six species of skinks, two species of cordylids, and one gerrhosaurid. We applied conventional and molecular cytogenetic methods, including C-banding, fluorescence in situ hybridization with probes specific for telomeric motifs and rDNA loci, and comparative genomic hybridization. The diploid chromosome numbers are rather conserved among these species, but the chromosome morphology, the presence of interstitial telomeric sequences, and the topology of rDNA loci vary significantly. Notably, XX/XY sex chromosomes were identified only in *Tiliqua scincoides*, where, in contrast to the X chromosome, the Y chromosome lacks accumulations of rDNA loci. We confirm that within the lizards of the scincoidean clade, sex chromosomes remained in a generally poor stage of differentiation.

## 1. Introduction

The lizard clade Scincoidea consists of four families: Scincidae, Xantusiidae, Gerrhosauridae, and Cordylidae, representing a highly diversified clade of squamate reptiles (currently around 1900 species), with a large morphological and ecological variability and nearly cosmopolitan distribution [1]. Despite their remarkable diversification, scincoidean lineages show rather similar cytogenetic traits [2,3,4]. Skinks (Scincidae) have relatively low diploid numbers (2n = 22 to 2n = 36 chromosomes). Their chromosomes can be categorized into macro- and microchromosomes [3,4]. Variability in diploid chromosome numbers was reported in the subfamily Scincinae, where 2n in most members vary between 26 and 36 [5,6,7]. The same is true for the skinks of the subfamily Lygosominae, where many species show diploid chromosome numbers of 2n = 30 or 2n = 32 [8], while few species show lower numbers, e.g., 2n = 28 in *Dasia vittata* [9]. In the subfamily Acontinae, which is a sister to all other skink lineages, the diploid chromosome numbers are low (2n = 22) [10]. The majority of the examined species in the families Cordylidae and Gerrhosauridae have diploid chromosome numbers of 2n = 34 [2]. In the family Xantusiidae, the diploid numbers vary from 2n = 30 to 2n = 34, apart from *Cricosaura typica*, which has a diploid chromosome number of only 2n = 24 [11,12].

The scincoidean lizards have been examined cytogenetically for decades, but sex chromosomes were reported only rarely. XX/XY sex chromosomes have been identified in a few skink species by C-banding, silver-staining, fluorescence in situ hybridization (FISH) with repetitive elements and rDNA loci, or by size differences between X and Y [7,13,14,15]. Heteromorphic XX/XY sex chromosomes were documented in *Oligosoma oliveri* [13; reported there as *Cyclodina oliveri*], *Saproscincus czechurai*, *Lampropholis* sp. [16], *Ctenotus rawlinsoni*, *Pseudemoia entrecasteauxii*, and *Pseudemoia pagenstecheri* [17]. In *Acritoscincus* (formery *Bassiana*) *duperreyi*, Shine et al. [18] identified XX/XY chromosomes by size differences between X and Y, which was later confirmed by C-banding, CGH and FISH with telomeric motifs [14], and isolation of a Y-specific molecular marker [19]. An interesting situation was found in the genus *Scincella*. Heteromorphic XX/XY sex chromosomes were identified in three American species [20,21] and X_1_X_1_X_2_X_2_/X_1_X_2_Y multiple sex chromosomes were found in a population of *Scincella lateralis*. Hedin et al. [22], based on the analysis of synaptonemal complexes, concluded that the X_1_X_1_X_2_X_2_/X_1_X_2_Y is derived from XX/XY by fusion of the Y chromosome with a macrochromosome. On the other hand, heteromorphic ZZ/ZW sex chromosomes were reported in the congeneric *Scincella melanosticta* from Thailand [23], representing the only reported case of female heterogamety in skinks.

Recently, two studies based on whole-genome sequencing and subsequent genomic coverage analysis identified shared X-specific gene content in two phylogenetically distant skink species, *Scincus scincus* [24] and *Eulamprus heatwolei* [25]. The X-specific exonic sequences of *S. scincus* were used to develop a qPCR-based method, which allowed to test the homology of sex chromosomes across 13 additional species of skinks covering most of the phylogenetic spectrum of the family Scincidae [24]. The same study revealed that the XX/XY sex chromosomes of skinks are poorly differentiated, but evolutionary stable for at least 85 million years [24]. The high age of the XX/XY sex determination system in skinks was independently supported by the analysis of the divergence of gametologs [25]. Recently, a Y-specific probe was designed from Illumina reads, derived from double-digest restriction-site associated DNA sequencing (ddRADseq), which accurately revealed the Y chromosome in the skink *Carinascincus ocellatus*, but not in the closely related *Liopholis whitii* [15]. Notably, heteromorphic ZZ/ZW sex chromosomes have been identified by conventional staining in the skink *Scincella melanosticta* [23], which would represent a rare case of sex chromosome turnover among skinks. ZZ/ZW sex chromosomes have been identified in the xantusiid *Xantusia henshawi* by restriction site-associated DNA sequencing (RADseq) [26]. A ZZ/ZW sex determination is also in accordance with the production of both sexes by facultative parthenogenesis in the xantusiid *Lepidophyma smithii* [27].

Sex determination is highly understudied in the families Gerrhosauridae and Cordylidae, and so far, ZZ/ZW sex chromosomes were identified only in the gerrhosaurid *Tracheloptychus petersi* by FISH with a probe for the rDNA loci, but not with comparative genome hybridization (CGH) [28]. The W chromosome of this species lacks the accumulation of rDNA loci linked to Z chromosomes.

In this study, we applied conventional (C-banding, karyogram preparation) and molecular cytogenetic (FISH with rDNA and telomeric probes, comparative genomic hybridization) methods to metaphases of, wherever possible, male and female specimens of nine species of the Scincoidea radiation (Table 1), in order to analyze their karyotype and to explore the presence of sex chromosomes.

## 2. Results

### 2.1. Acontias percivali

**Figure 1:**
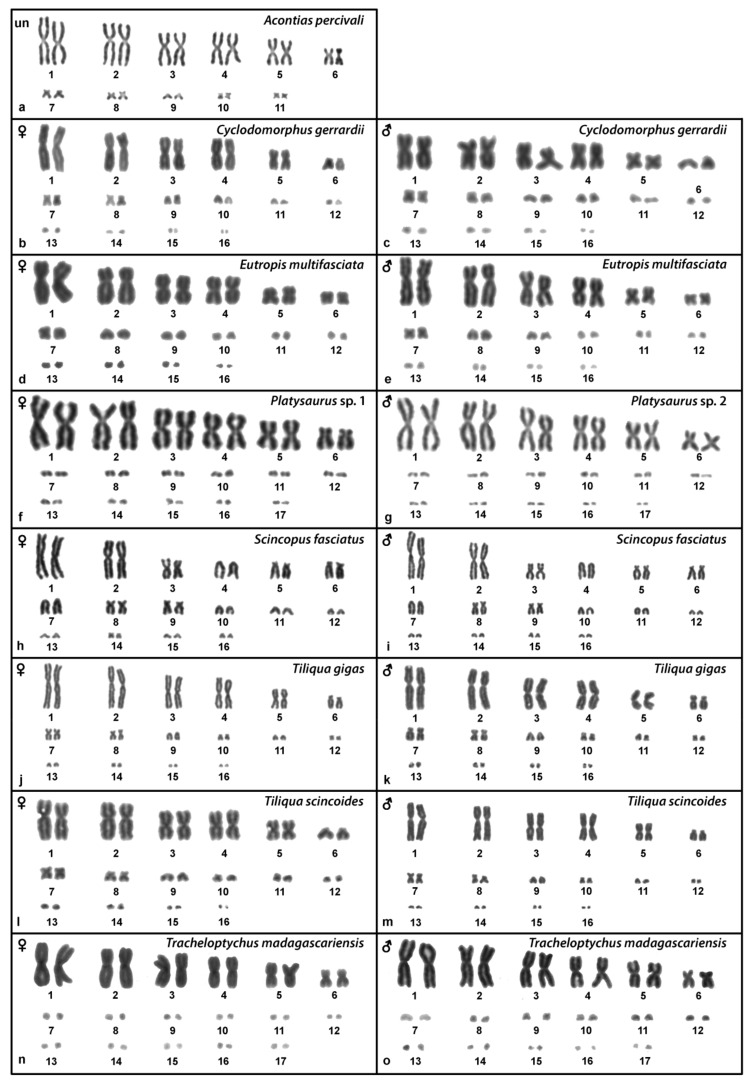
Giemsa-stained karyograms of *Acontias percivali* (**a**), *Cyclodomorphus gerrardii* (**b**,**c**), *Eutropis multifasciata* (**d**,**e**), *Platysaurus* sp. 1 (**f**), *Platysaurus* sp. 2 (**g**), *Scincopus fasciatus* (**h**,**i**), *Tiliqua gigas* (**j**,**k**), *Tiliqua scincoides* (**l**,**m**), and *Tracheloptychus madagascariensis* (**n**,**o**). UN: sex not known.

**Figure 2:**
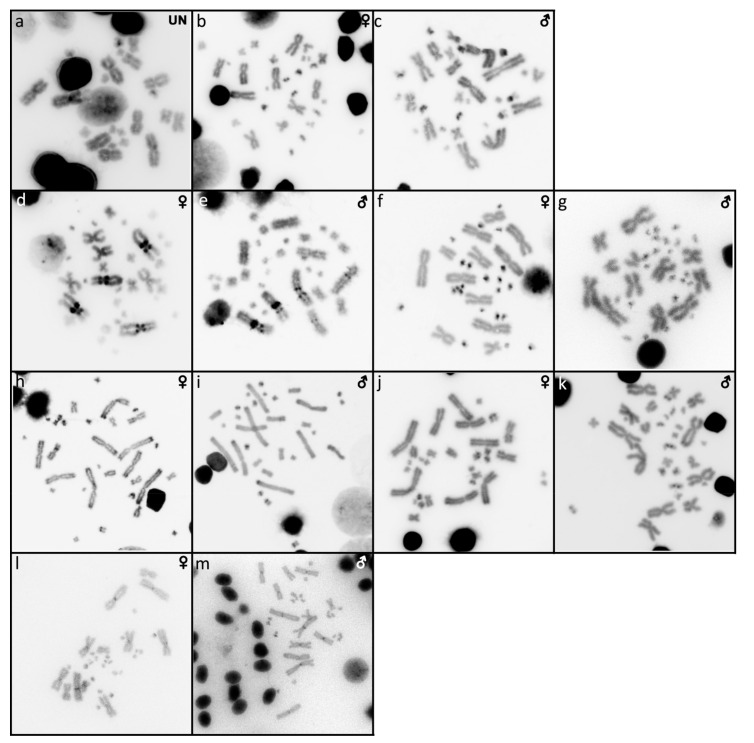
C-banded metaphases of *Acontias percivali* (**a**), *Cyclodomorphus gerrardii* (**b**,**c**), *Eutropis multifasciata* (**d**,**e**), *Platysaurus* sp. 1 (**f**), *Platysaurus* sp. 2 (**g**), *Tiliqua gigas* (**h**,**i**), *Tiliqua scincoides* (**j**,**k**), and *Tracheloptychus madagascariensis* (**l**,**m**). UN: sex not known.

**Figure 3:**
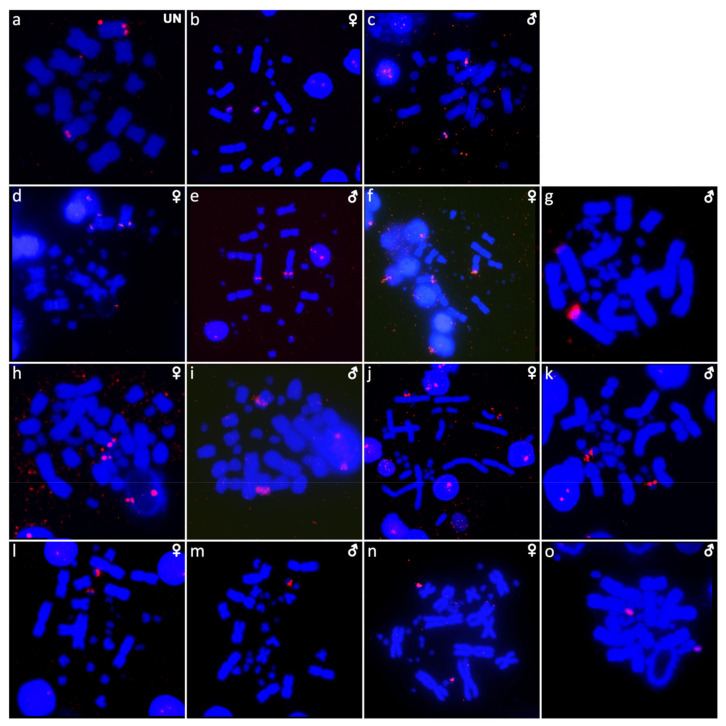
In situ hybridization with probe for 18S/28S rDNA sequences in *Acontias percivali* (**a**), *Cyclodomorphus gerrardii* (**b**,**c**), *Eutropis multifasciata* (**d**,**e**), *Platysaurus* sp. 1 (**f**), *Platysaurus* sp. 2 (**g**), *Scincopus fasciatus* (**h**,**i**), *Tiliqua gigas* (**j**,**k**), *Tiliqua scincoides* (**l**,**m**), and *Tracheloptychus madagascariensis* (**n**,**o**). UN: sex not known.

**Figure 4:**
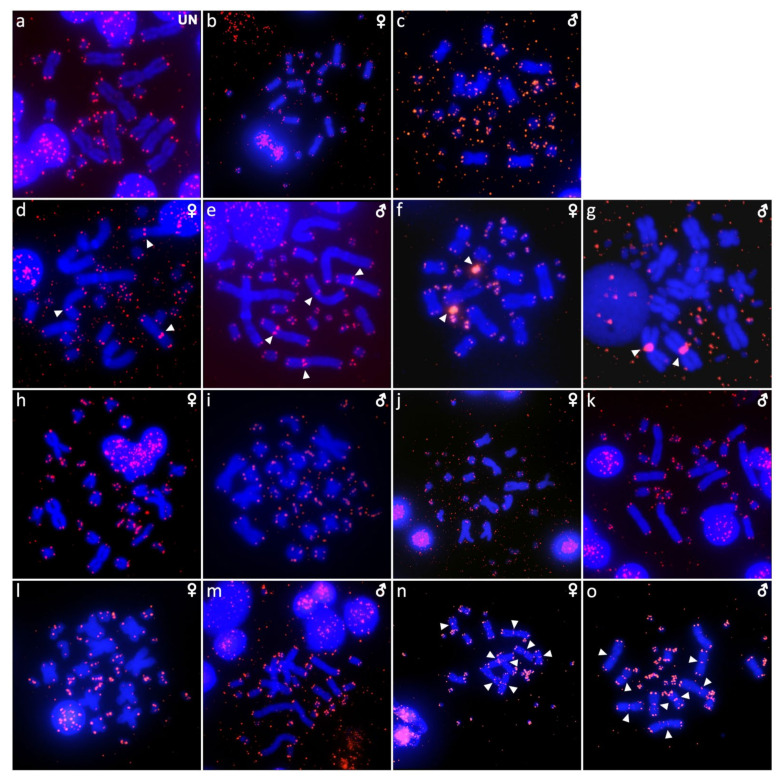
In situ hybridization with probe for telomeric sequences in *Acontias percivali* (**a**), *Cyclodomorphus gerrardii* (**b**,**c**), *Eutropis multifasciata* (**d**,**e**), *Platysaurus* sp. 1 (**f**), *Platysaurus* sp. 2 (**g**), *Scincopus fasciatus* (**h**,**i**), *Tiliqua gigas* (**j**,**k**), *Tiliqua scincoides* (**l**,**m**), and *Tracheloptychus madagascariensis* (**n**,**o**). UN: sex not known.

The karyotype consists of 2n = 22 chromosomes (Figure 1a), which is in line with the observations of Gordon [10]. We were not able to identify the sex of the studied specimen. Heterochromatic blocks are present in the centromeric region of a pair of macrochromosomes and a pair of microchromosomes (Figure 2a). FISH with the rDNA probe revealed signals at the terminal position of a pair of macrochromosomes, corresponding to the chromosome pair 3 or 4 (but we cannot safely conclude since both pairs have similar morphology) (Figure 3a). Telomeric sequences are visible at the terminal position of all chromosomes (Figure 4a).

### 2.2. Cyclodomorphus gerrardii

The karyotype consists of 2n = 32 chromosomes, as previously described in Donnellan [16] (Figure 1b,c). Both male and female specimens were examined. C-banding revealed heterochromatin in several microchromosomes (Figure 2b,c). FISH with the rDNA probe revealed signals in one pair of microchromosomes (Figure 3b,c). Telomeric sequences are present at terminal positions of all chromosomes (Figure 4b,c).

### 2.3. Eutropis multifasciata

**Figure 5:**
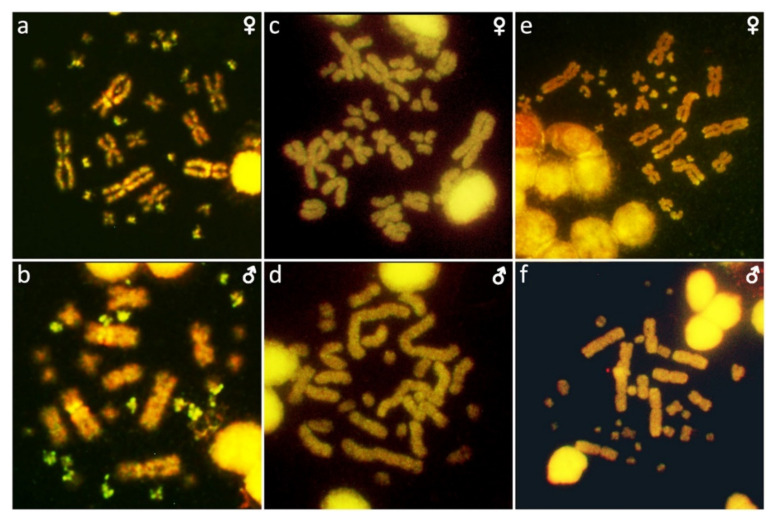
Comparative genome hybridization in *Eutropis multifasciata* (**a**,**b**), *Scincopus fasciatus* (**c**,**d**), and *Tiliqua gigas* (**e**,**f**).

The karyotype consists of 2n = 32 chromosomes, as previously described by de Smet [29] (Figure 1d,e). Both male and female specimens were examined. Heterochromatic blocks can be found in the pericentromeric regions of three macrochromosome pairs (Figure 2d,e). Signals of rDNA loci were revealed on chromosome pair 1 (Figure 3d,e). Telomeric sequences are located at terminal positions of all chromosomes, and additionally in the pericentromeric region on chromosome pairs 3 and 4 (Figure 4d,e). CGH revealed no sex-specific differences (Figure 5a,b).

### 2.4. Platysaurus sp. 1

The karyotype consists of 2n = 34 chromosomes, with 12 of them being macrochromosomes (Figure 1f). A single female specimen was examined. C-banding revealed heterochromatic regions on all microchromosomes (Figure 2f). FISH with the rDNA probe revealed signals at the terminal position in chromosome pair 3 (Figure 3f). Telomeric sequences are found at terminal positions on all chromosomes. One pair of microchromosomes shows accumulation of interstitial telomeric repeats (ITRs) (Figure 4f).

### 2.5. Platysaurus sp. 2

The karyotype consists of 2n = 34 chromosomes (12 macrochromosomes) (Figure 1g). A single male specimen was examined. Heterochromatic blocks are found on microchromosomes (Figure 2g). FISH with rDNA revealed a signal at the terminal position of the chromosome pair 3 (Figure 3g). Telomeric sequences at terminal positions are present, but poorly visible in some chromosomes. A prominent telomeric signal is present at the centromere of one pair of macrochromosomes (Figure 4g).

### 2.6. Scincopus fasciatus

The karyotype consists of 2n = 32 chromosomes with several telocentric and acrocentric pairs (Figure 1h,i). Both male and female specimens were examined. C-banding did not reveal any heterochromatic block. Signals of rDNA were visible on a submetacentric pair of macrochromosomes, corresponding to chromosome pair 5 or 6 (but we cannot safely conclude since both pairs have similar morphology) (Figure 3h,i). Telomeric sequences were found at terminal positions of all chromosomes and interstitial telomeric repeats (ITRs) were absent (Figure 4h,i). Sex-specific differences were not detected by CGH (Figure 5c,d).

### 2.7. Tiliqua gigas

The karyotype consists of 2n = 32 chromosomes (Figure 1j,k), as previously reported by Donnellan [16]. Both male and female specimens were examined. Heterochromatic regions are visible at two pairs of macrochromosomes as well as several microchromosomes (Figure 2h,i). Signals of rDNA were present on one pair of microchromosomes (Figure 3j,k). Telomeric sequences were found at the terminal regions of all chromosomes (Figure 4j,k). CGH revealed no sex-specific differences (Figure 5e,f).

### 2.8. Tiliqua scincoides

The karyotype consists of 2n = 32 chromosomes (Figure 1l,m), as previously described by de Smet [29]. Both male and female specimens were examined. Heterochromatic regions are present in several microchromosomes (Figure 2j,k). FISH with the rDNA probe showed signals on two microchromosomes in females, but only on a single microchromosome in the male (Figure 3l,m). Telomeric sequences are present at the terminal position of all chromosomes (Figure 4l,m).

### 2.9. Tracheloptychus madagascariensis

The karyotype consists of 2n = 34 chromosomes (Figure 1n,o), with 12 of them being macrochromosomes, as previously described by Odierna et al. [2]. Both male and female specimens were examined. Heterochromatin is present in the centromeric region of four pairs of macrochromosomes as well as in several microchromosomes (Figure 2l,m). Signals of the rDNA probe are visible on a pair of microchromosomes (Figure 3n,o). Telomeric sequences are present at the terminal position in all chromosomes and ITRs are present in four pairs of macrochromosomes (Figure 4n,o).

## 3. Discussion

Except for *Acontias percivali* with 2n = 22 chromosomes (Figure 1a), all other studied species showed a low variation in the diploid chromosome numbers, having karyotypes with 2n = 32 or 2n = 34 chromosomes (Figure 1). Our results are following previous cytogenetic studies, which showed that karyotypes with 2n = 28 to 2n = 34 are mostly reported in skinks, cordylids, and gerrhosaurids [3]. Nevertheless, chromosome morphology (mainly centromere position and chromosome size) varied significantly among species with the same chromosome number (Figure 1). We speculate that chromosomal rearrangements (including inversions) have commonly occurred during the karyotype evolution in Scincoidea, in contrast to other reptilian lineages such as turtles, where chromosome morphology seems to be rather unchanged in the long term [30].

Heterochromatic regions were detected in the centromeric and pericentromeric regions, mainly on macrochromosomes, in *Eutropis multifasciata* (three pairs), *Tiliqua gigas* (two pairs), and *Tracheloptychus madagascariensis* (four pairs) (Figure 2). Heterochromatic blocks were detected in several microchromosomes in *Acontias percivali*, *Cyclodomorphus gerrardii*, *Eutropis multifasciata*, *Platysaurus* sp. 1, *Platysaurus* sp. 2, *Tiliqua gigas*, *Tiliqua scincoides*, and *Tracheloptychus madagascariensis* (Figure 2). Notably, heterochromatic blocks were not detected in *Scincopus fasciatus.* A clear phylogenetic pattern was not detected in the distribution of heterochromatic regions among scincoidean species.

The comparative coverage analysis of Illumina DNAseq reads revealed homologous XX/XY sex chromosomes in the distantly related skinks *Scincus scincus* [24], *Eulamprus heatwolei*, and *Eulamprus tympanum* [25]. A qPCR-based method, which is based on the comparison of copies of X-specific genes between sexes supported the homology of XX/XY sex chromosomes across 13 species of skinks. The same technique was used in all skinks in the current paper, except for *Acontias percivali*, to validate the sex of each species. Therefore, we are aware that all the skinks studied here, except *Acontias percivali*, have homologous XX/XY sex chromosomes. However, the sex chromosomes were detected by cytogenetic methods only in *Tiliqua scincoides* (Figure 3l,m). It seems that these highly evolutionary stable sex chromosomes are poorly differentiated and mostly cryptic in the majority of the skink species. In *Tiliqua scincoides*, the probe specific for the rDNA loci hybridized in a single microchromosome in males, but in two microchromosomes in females (Figure 3l,m). We conclude that the rDNA loci seem to be located on the X chromosome, but missing on the Y, as was previously revealed in the distantly related *Scincus scincus* [24]. *Scincopus fasciatus*, which belongs to the same subfamily as *S. scincus* (Scincinae), is missing this sex-specific distribution of rDNA loci. Ribosomal DNA loci show sex-specific accumulations in many taxa, including the gerrhosaurid *Tracheloptychus petersi*, where they are located on a pair of autosomes, and additionally, on the Z chromosome, but missing on the W chromosome [28]. In the closely related *Tracheloptychus madagascariensis*, rDNA loci are present in a pair of microchromosomes in both sexes (Figure 3o,p), which could be homologous to the sex chromosomes of *T. petersi*. Odierna et al. [2] observed in *T. madagascariensis* the same distribution of active rDNA loci, visualized by silver staining. Notably, we detected rDNA loci in a single macrochromosome in the male of *Platysaurus* sp. 2, but we could not distinguish if the single signal reflects an autosomal polymorphism or a sex chromosome. Additional specimens from both sexes are needed to further investigate the sex determination system in this species.

Interspecific differences in the sex-linkage of rDNA loci are common in reptiles. In Australasian side-necked turtles, sex-specific differences are present in *Chelodina novaeguineae*, *Chelodina reimanni*, *Elseya novaeguineae*, and the hybrid *Emydura subglobosa* × *Elseya novaeguineae*, but not in *Chelodina expansa*, *Chelodina rugosa*, *Chelodina mccordi*, and *Emydura macquarii krefftii* [31]. In the spiny softshell turtle *Apalone spinifera*, accumulations of rDNA loci are present on both the Z and W, but the block is much bigger on the W [32]. This difference in copy number of rDNA loci was further demonstrated by qPCR in nine tested trionychid species, but in *Lissemys punctata punctata*, male and female had equal number of copies of rDNA loci, and in *Lissemys punctata andersoni*, females even had less copies of rDNA loci than males [33]. Not only sex-specificity, but also the overall distribution of rDNA loci in the karyotype vary greatly among species. rDNA loci are present on macrochromosomes in *Acontias percivali*, *Eutropis multifasciata*, *Scincopus fasciatus*, *Platysaurus* sp. 1, and *Platysaurus* sp. 2, but on microchromosomes in *Cyclodomorphus gerrardii*, *Tiliqua gigas*, *Tiliqua scincoides*, and *Tracheloptychus madagascariensis*. Currently, we cannot conclude if the variability in the position of rDNA loci between species correspond to homologous genomic regions reshuffled by chromosomal rearrangements (e.g., fusions/fissions), or independent emergence of amplification of rDNA loci. The variability in chromosome position and number of repeats has been previously reported in other vertebrate lineages and it is often linked to the high mobility and intrinsic recombinational instability of rDNA loci [34,35].

Comparative genomic hybridization is another molecular cytogenetic tool commonly used to identify sex chromosomes. This method uncovered the W chromosome in several reptiles, including the agamid *Pogona vitticeps*, the acrochordid snake *Acrochordus javanicus*, several species of the chameleon genus *Furcifer* and of monitor lizards, as well as the Y chromosome in the Australasian side-necked turtles *Chelodina expansa*, *Chelodina novaeguineae*, and *Elseya novaeguineae*, in the legless geckos of the genus *Lialis* and in the Cuvier’s Madagascar swift *Oplurus cuvieri* [31,36,37,38,39,40,41]. On the contrary, comparative genomic hybridization did not identify the W chromosome in *Tracheloptychus petersi* [28] and the Y chromosomes in *Scincus scincus*, *Tropidophorus baconi* [24], *Eutropis multifasciata*, *Scincopus fasciatus*, and *Tiliqua gigas* (Figure 5). Genome sequencing revealed that the X-specific region in the skinks *Scincus scincus* [24] and *Eulamprus heatwolei* [25] makes up only a small portion of the X chromosome, and it is probably beyond the detection efficiency of the method. Despite the long-term stability of their XX/XY sex determination system (estimated to 79–116 My) [24,25], skinks seem to have poorly differentiated sex chromosomes. Although the canonical model of sex chromosome evolution [42,43] predicts that sex chromosomes should differentiate over-time, which leads to the loss of functional genes, accumulation of repetitive elements and heterochromatinization of Y/W, skinks consist a rare example among vertebrates, where sex chromosomes remained in a poorly differentiated stage in the long term. In addition, poorly differentiated sex chromosomes tend to be prone to sex chromosome turnovers [44,45], but skinks constitute a notable case that even poorly differentiated sex chromosomes can be stable for a long evolutionary time. We believe that skinks are a magnificent group to enrich our knowledge and deserve further attention to explore the drivers of sex chromosome evolution.

Despite the fact that karyograms revealed extensive differences in chromosome morphology between species, interstitial telomeric repeats were documented only in the skink *Eutropis multifasciata*, the gerrhosaurid *Tracheloptychus madagascariensis*, and the cordylids *Platysaurus* sp. 1 and *Platysaurus* sp. 2 (Figure 4). Interstitial telomeric repeats are common in squamate reptiles [46], and can be remnants of interchromosomal rearrangements, or the activity of transposable elements [47,48,49,50,51]. ITRs can be part of satellite motifs, and occasionally are present in the heterochromatic regions of sex chromosomes [31,52,53,54]. Data on the distribution of ITRs are still scarce, but ITRs were not detected in the sex chromosomes of the studied species from the scincoidean clade. Notably, the variability in the topology of ITRs has been detected in the closely related species *Tracheloptychus madagascariensis* (present study) and *Tracheloptychus petersi* [28], despite the fact that both species share similar karyotypes.

In summary, the lizards of the clade Scincoidea have karyotypes with little variability in diploid chromosome number and chromosome variability. Nevertheless, molecular cytogenetic methods revealed extensive variability in the topology of rDNA loci and interstitial telomeric repeats among species. Despite the fact that previous studies revealed an extreme homology of sex chromosomes across skinks based on genomic and molecular methods [24,25], by cytogenetic methods we identified XX/XY sex chromosomes only in *Tiliqua scincoides* indicating that the sex chromosomes remained at a generally poor stage of differentiation in skinks, and perhaps in other lineages of the clade Scincodea. Future studies should focus on revealing the sex chromosome gene content by genomic methods in phylogenetically informative species of the scincoidean megadiversity. Prominent topics of research should be the validation of the ZZ/ZW sex determination in the skink *Scincella melanosticta*, the identification of the sex determination mode in cordylids, and the test of homology of ZZ/ZW sex determination systems between gerrhosaurids and xantusiids.

## 4. Materials and Methods

### 4.1. Samples and Species Verification

We studied nine species from three families of the clade Scincoidea: *Platysaurus* sp. 1, *Platysaurus* sp. 2 (Cordylidae), *Tracheloptychus madagascariensis* (Gerrhosauridae), *Acontias percivali, Cyclodomorphus gerrardii, Eutropis multifasciata, Scincopus fasciatus, Tiliqua gigas*, and *Tiliqua scincoides* (Scincidae) (Table 1). We studied both male and female individuals in all species, apart from *Acontias percivali*, *Platysaurus* sp. 1, and *Platysaurus* sp. 2, where either only a single specimen was available or the sex could not be identified by external morphology or everting hemipenes (i.e., the male-specific reproductive organs) by palpation. For skinks from the subfamilies Lygosominae and Scincinae, the sex was verified by our recently developed qPCR-based molecular sexing method (Kostmann et al. 2021a).

### 4.2. Chromosome Preparation and Staining

Whole blood cell cultures were prepared with fresh blood, incubated in DMEM medium (Gibco, Thermo Fisher Scientific Inc., Waltham, MA, USA), enriched with 10% fetal bovine serum (Gibco, Thermo Fisher Scientific Inc., Waltham, MA, USA), 1% penicillin/streptomycin solution (Gibco, Thermo Fisher Scientific Inc., Waltham, MA, USA), 1% L-glutamine solution (Sigma-Aldrich, St. Louis, MO, USA), 3% phytohemagglutinin (Gibco, Thermo Fisher Scientific Inc., Waltham, MA, USA), and 1% lipopolysaccharide solution (Sigma-Aldrich, St. Louis, MO, USA). After one week of incubation at 30 °C, the cells were subsequently treated with 35 µL colchemid solution (Sigma-Aldrich, St. Louis, MO, USA) for 3.5 h and 0.075 M KCl for 30 min. Fixation was done by four rounds of treatment with 3:1 methanol/acetic acid solution at 4 °C. Chromosomal spreads were stained with 6% Giemsa solution for karyogram preparation. Heterochromatic regions were visualized by C-banding, following the protocol of Sumner [55] with slight modifications. Chromosomal spreads were dried at 60 °C for 1 h and subsequently incubated in 0.2 N HCl for 40 min, 5% Ba(OH)_2_ at 45 °C for 5 min, saline–sodium citrate solution (2 × SSC) at 60 °C for 1 h, washed with distilled water and air-dried. Afterwards, the chromosomal spreads were stained with Fluoroshield containing DAPI (4′,6-diamidino-2-phenylindole; Sigma-Aldrich, St. Louis, MO, USA).

### 4.3. Fluorescence In Situ Hybridization with Probes for rDNA Loci (18S/28S) and Telomeric Motifs

We prepared the probe for the rDNA loci from a plasmid (pDmr.a 51#1) with an 11.5-kb insert encoding the 18S and 28S ribosomal units of *Drosophila melanogaster* [56], labelled by nick-translation with dUTP-biotin. The telomeric probe was prepared through PCR, using the primers (TTAGGG)_5_ and (CCCTAA)_5_, without a DNA template, according to Ijdo et al. [57]. Both probes were precipitated with salmon sperm and sodium acetate in ethanol at −20 °C overnight. After centrifugation and drying, the pellet was dissolved in a hybridization solution (50% formamide in 2 × SSC). For the fluorescence in situ hybridization, the chromosomal spreads were hydrated in 2 × SSC for 5 min at room temperature, followed by RNase treatment for 1 h at 37 °C, washing in 2 × SSC for 5 min at room temperature (3 times), pepsin treatment for 10 min at 37 °C, washing in PBS for 5 min at room temperature (2 times) and 1% formaldehyde in PBS for 10 min. Dehydration through an ethanol series (70%, 85%, 100%), denaturation in 70% formamide for 4 min at 75 °C, followed by dehydration in ethanol series. Before hybridization, the probes were incubated at 75 °C for 6 min, followed by 10 min at −20 °C. The probe was incubated on the chromosomal spread at 37 °C overnight. Post-hybridization washes were performed in 50% formamide for 5 min at 37 °C (3 times), 2 × SSC for 5 min at room temperature (2 times), and washed in 4 × SSC/0.05% Tween20 (Sigma). Chromosomal spreads were treated with 4 × SSC/5% blocking reagent (Roche, Basel, Switzerland) for 45 min at 37 °C. Incubation with 4 × SSC/5% blocking reagent with avidin-FITC (Vector Laboratories, Burlingame, CA, USA) for 30 min at 37 °C was followed by two consecutive treatments with a modified system of avidin-FITC/biotinylated anti-avidin signal enhancement (Vector Laboratories, Burlingame, CA, USA). Chromosomal spreads were stained by Fluoroshield with DAPI (Sigma-Aldrich, St. Louis, MO, USA).

### 4.4. Comparative Genomic Hybridization

Male DNA was labeled with dUTP-biotin and female DNA was labeled with dUTP–digoxigenin using a Nick Translation Kit (Abbott Laboratories, Lake Bluff, IL, USA). The labelled DNA from both sexes was co-precipitated with salmon sperm and sodium acetate (3M) in ethanol for 1 h at −80 °C. After centrifuging and drying, the probe was dissolved in hybridization mix (50% formamide in 2 × SSC). Before hybridization, the probe was heated to 75 °C for 6 min, followed by 10 min at −20 °C. Chromosomal spreads before hybridization were treated in the same way as described above for the FISH experiments, but hybridization time was increased to two nights. Post-hybridization washes were done in 50% formamide at 37 °C for 5 min, repeated three times. The slides were washed three times in 2 × SSC for 5 min and once in 4 × SSC/0.1% Tween20 for 30 s, followed by 4 × SSC/5% blocking reagent for 30 min at 37 °C. The slides were incubated with 4 × SSC/5% blocking reagent, containing avidin-FITC and anti-digoxigenin–rhodamine for 30 min at 37 °C, they were washed three times with 4 × SSC/0.1% Tween20, dehydrated through an ethanol series, and stained with Fluoroshield with DAPI (Sigma-Aldrich, St. Louis, MO, USA). Comparative genome hybridization in *Eutropis multifasciata*, *Scincopus fasciatus*, and *Tiliqua gigas* (Figure 5).

### 4.5. Microscopy and Image Analyses

For each specimen and method, we captured at least 10 images by either a Zeiss Axio Imager Z2 (Zeiss, Oberkochen, Germany) equipped with an automatic Metafer-MSearch scanning platform and a CoolCube 1 b/w digital camera (MetaSystems, Altlussheim, Germany) or an Olympus Provis AX70 fluorescence microscope equipped with a DP30BW digital camera (Olympus, Tokyo, Japan).

## Figures and Tables

**Table 1:** Species analyzed in the present study.

Species	Family	Subfamily	Sex		
			Male	Female	Unknown
*Platysaurus* sp. 1	Cordylidae	Platysaurinae	-	1	-
*Platysaurus* sp. 2	Cordylidae	Platysaurinae	1	-	-
*Tracheloptychus madagascariensis*	Gerrhosauridae	Zonosaurinae	1	1	-
*Acontias percivali*	Scincidae	Acontinae	-	-	1
*Cyclodomorphus gerrardii*	Scincidae	Lygosominae	2	2	-
*Eutropis multifasciata*	Scincidae	Lygosominae	2	1	-
*Tiliqua gigas*	Scincidae	Lygosominae	5	3	-
*Tiliqua scincoides*	Scincidae	Lygosominae	1	3	-
*Scincopus fasciatus*	Scincidae	Scincinae	1	1	-

## Data Availability

All data are provided in the current manuscript.

## References

[1] Uetz P., Freed P., Aguilar R., Hošek J. The Reptile Database. http://www.reptile-database.org.

[2] Odierna G., Canapa A., Andreone F., Aprea G., Barucca M., Capriglione T., Olmo E. (2002). A phylogenetic analysis of Cordyliformes (Reptilia: Squamata): Comparison of molecular and karyological data. Mol. Phylogenetics Evol..

[3] Olmo E., Signorino G. Chromorep: A Reptile Chromosomes Database. http://chromorep.univpm.it.

[4] Giovannotti M., Caputo V., O’Brien P., Lovell F., Trifonov V., Cerioni P.N., Olmo E., Ferguson-Smith M., Rens W. (2009). Skinks (Reptilia: Scincidae) have highly conserved karyotypes as revealed by chromosome painting. Cytogenet. Genome Res..

[5] Deweese J.E., Wright J.W. (1970). A preliminary karyological analysis of scincid lizards. Mamm. Chromosome Newsl..

[6] Branch W.R. (1980). Chromosome morphology of some reptiles from Oman and adjacent territories. J. Oman Stud. Rep..

[7] Caputo V., Odierna G., Aprea G. (1994). A chromosomal study of *Eumeces* and *Scincus*, primitive members of the Scincidae (Reptilia, Squamata). Bolletino Zool..

[8] King M. (1973). Karyotypic studies of some Australian Scincidae (Reptilia). Aust. J. Zool..

[9] Ota H., Hikida T., Matsui M., Hasegawa M., Labang D., Nabhitabhata J. (1996). Chromosomal variation in the scincid genus *Mabuya* and its arboreal relatives (Reptilia: Squamata). Genetica.

[10] Gordon D.H., Haacke W.D., Jacobsen N.H.G. (1989). Chromosomal studies of relationships in Gekkonidae, Chamaeleonidae and Scincidae in South Africa. J. Herpetol. Assoc. Afr..

[11] Hass C.A., Hedges S.B. (1992). Karyotype of the Cuban lizard *Cricosaura typica* and its implications for xantusiid phylogeny. Copeia.

[12] Bezy R.L. (1972). Karyotypic variation and evolution of the lizards in the family Xantusiidae. Los Angeles County Mus. Contr. Sci..

[13] Hardy G.S. (1979). The karyotypes of two scincid lizards, and their bearing on relationships in genus *Leiolopisma* and its relatives (Scincidae: Lygosominae). N. Z. J. Zool..

[14] Matsubara K., O’Meally D., Azad B., Georges A., Sarre S.D., Graves J., Matsuda Y., Ezaz T. (2016). Amplification of microsatellite repeat motifs is associated with the evolutionary differentiation and heterochromatinization of sex chromosomes in Sauropsida. Chromosoma.

[15] Hill P., Shams F., Burridge C., Wapstra E., Ezaz T. (2021). Differences in homomorphic sex chromosomes are associated with population divergence in sex determination in *Carinascincus ocellatus* (Scincidae: Lygosominae). Cells.

[16] Donnellan S. (1991). Chromosomes of Australian lygosomine skinks (Lacertilia: Scincidae). Genetica.

[17] Hutchinson M.N., Donnellan S. (1992). Taxonomy and genetic variation in the Australian lizards of the genus *Pseudemoia* (Scincidae: Lygosominae). J. Nat. Hist..

[18] Shine R., Elphick M.J., Donnellan S. (2002). Co-occurrence of multiple, supposedly incompatible modes of sex determination in a lizard population. Ecol. Lett..

[19] Quinn A.E., Radder R.S., Sarre S.D., Georges A., Ezaz T., Shine R. (2009). Isolation and development of a molecular sex marker for *Bassiana duperreyi*, a lizard with XX/XY sex chromosomes and temperature-induced sex reversal. Mol. Genet. Genom..

[20] Wright J.W. (1973). Evolution of the X_1_X_2_Y sex chromosome mechanism in the scincid lizard *Scincella laterale* (Say). Chromosoma.

[21] Castiglia R., Bezerra A., Flores-Villela O., Annesi F., Muñoz A., Gornung E. (2013). Comparative cytogenetics of two species of ground skinks: *Scincella assata* and *S. cherriei* (Squamata: Scincidae: Lygosominae) from Chiapas, Mexico. Acta Herpetol..

[22] Hedin M.C., Sudman P.D., Greenbaum I.F., Sites J.W. (1990). Synaptonemal complex analysis of sex chromosome pairing in the common ground skink, *Scincella lateralis* (Sauria, Scincidae). Copeia.

[23] Patawang I., Chuaynkern Y., Supanuam P., Maneechot N., Pinthong K., Tanomtong A. (2018). Cytogenetics of the skinks (Reptilia, Scincidae) from Thailand; IV: Newly investigated karyotypic features of *Lygosoma quadrupes* and *Scincella melanosticta*. Caryologia.

[24] Kostmann A., Kratochvíl L., Rovatsos M. (2021). Poorly differentiated XX/XY sex chromosomes are widely shared across skink radiation. Proc. R. Soc. B Biol. Sci..

[25] Cornejo-Páramo P., Dissanayake D., Lira-Noriega A., Martínez-Pacheco M.L., Acosta A., Ramírez-Suástegui C., Méndez-De-La-Cruz F.R., Székely T., Urrutia A.O., Georges A. (2020). Viviparous reptile regarded to have temperature-dependent sex determination has old XY chromosomes. Genome Biol. Evol..

[26] Nielsen S.V., Pinto B.J., Guzmán-Méndez I.A., Gamble T. (2020). First report of sex chromosomes in night lizards (Scincoidea: Xantusiidae). J. Hered..

[27] Kratochvíl L., Vukić J., Červenka J., Kubička L., Pokorná M.J., Kukačková D., Rovatsos M., Piálek L. (2020). Mixed-sex offspring produced via cryptic parthenogenesis in a lizard. Mol. Ecol..

[28] Kostmann A., Kratochvíl L., Rovatsos M. (2020). First report of sex chromosomes in plated lizards (Squamata: Gerrhosauridae). Sex. Dev..

[29] De Smet W. (1981). Description of the orcein stained karyotypes of 36 lizard species (Lacertilia, Reptilia) belonging to the families Teiidae, Scincidae, Lacertidae, Cordylidae and Varanidae (Autarchoglossa). Acta. Zool. Pathol. Antverp..

[30] Clemente L., Mazzoleni S., Pensabene Bellavia E., Augstenová B., Auer M., Praschag P., Protiva T., Velenský P., Wagner P., Fritz U. (2020). Interstitial telomeric repeats are rare in turtles. Genes.

[31] Mazzoleni S., Augstenová B., Clemente L., Auer M., Fritz U., Praschag P., Protiva T., Velenský P., Kratochvíl L., Rovatsos M. (2020). Sex is determined by XX/XY sex chromosomes in Australasian side-necked turtles (Testudines: Chelidae). Sci. Rep..

[32] Badenhorst D., Stanyon R., Engstrom T., Valenzuela N. (2013). A ZZ/ZW microchromosome system in the spiny softshell turtle, *Apalone spinifera*, reveals an intriguing sex chromosome conservation in Trionychidae. Chromosome Res..

[33] Rovatsos M., Praschag P., Fritz U., Kratochvšl L. (2017). Stable Cretaceous sex chromosomes enable molecular sexing in softshell turtles (Testudines: Trionychidae). Sci. Rep..

[34] Porter C.A., Hamilton M.J., Sites J.W., Baker R.J. (1991). Location of ribosomal DNA in chromosomes of squamate reptiles: Systematic and evolutionary implications. Herpetologica.

[35] Stults D.M., Killen M.W., Pierce H.H., Pierce A.J. (2007). Genomic architecture and inheritance of human ribosomal RNA gene clusters. Genome Res..

[36] Ezaz T., Quinn A.E., Miura I., Sarre S.D., Georges A., Graves J. (2005). The dragon lizard *Pogona vitticeps* has ZZ/ZW micro-sex chromosomes. Chromosome Res..

[37] Matsubara K., Sarre S.D., Georges A., Matsuda Y., Graves J.A.M., Ezaz T. (2014). Highly differentiated ZW sex microchromosomes in the Australian *Varanus* species evolved through rapid amplification of repetitive sequences. PLoS ONE.

[38] Altmanová M., Rovatsos M., Kratochvíl L., Johnson Pokorná M. (2016). Minute Y chromosomes and karyotype evolution in Madagascan iguanas (Squamata: Iguania: Opluridae). Biol. J. Linn. Soc..

[39] Rovatsos M., Johnson Pokorná M., Altmanová M., Kratochvíl L. (2016). Mixed-up sex chromosomes: Identification of sex chromosomes in the X_1_X_1_X_2_X_2_/X_1_X_2_Y system of the legless lizards of the genus *Lialis* (Squamata: Gekkota: Pygopodidae). Cytogenet. Genome Res..

[40] Rovatsos M., Altmanová M., Johnson Pokorná M., Augstenová B., Kratochvíl L. (2017). Cytogenetics of the Javan file snake (*Acrochordus javanicus*) and the evolution of snake sex chromosomes. J. Zool. Syst. Evol. Res..

[41] Rovatsos M., Altmanová M., Augstenová B., Mazzoleni S., Velenský P., Kratochvíl L. (2019). ZZ/ZW sex determination with multiple neo-sex chromosomes is common in Madagascan chameleons of the genus *Furcifer* (Reptilia: Chamaeleonidae). Genes.

[42] Rice W.R. (1996). Evolution of the Y sex chromosome in animals. BioScience.

[43] Charlesworth B., Charlesworth D. (2000). The degeneration of Y chromosomes. Philos. Trans. R. Soc. B: Biol. Sci..

[44] Perrin N. (2009). Sex reversal: A fountain of youth for sex chromosomes?. Evolution.

[45] Jeffries D.L., Lavanchy G., Sermier R., Sredl M.J., Miura I., Borzée A., Barrow L.N., Canestrelli D., Crochet P.-A., Dufresnes C. (2018). A rapid rate of sex-chromosome turnover and non-random transitions in true frogs. Nat. Commun..

[46] Rovatsos M., Kratochvíl L., Altmanová M., Johnson Pokorná M. (2015). Interstitial telomeric motifs in squamate reptiles: When the exceptions outnumber the rule. PLoS ONE.

[47] Bolzán A.D., Bianchi M.S. (2006). Telomeres, interstitial telomeric repeat sequences, and chromosomal aberrations. Mutat. Res. Mutat. Res..

[48] Ruiz-Herrera A., Nergadze S.G., Santagostino M., Giulotto E. (2008). Telomeric repeats far from the ends: Mechanisms of origin and role in evolution. Cytogenet. Genome Res..

[49] Aksenova A.Y., Greenwell P.W., Dominska M., Shishkin A.A., Kim J.C., Petes T.D., Mirkin S.M. (2013). Genome rearrangements caused by interstitial telomeric sequences in yeast. Proc. Natl. Acad. Sci. USA.

[50] Bolzán A.D. (2017). Interstitial telomeric sequences in vertebrate chromosomes: Origin, function, instability and evolution. Mutat. Res. Mutat. Res..

[51] Shubernetskaya O., Skvortsov D., Dontsova O., Kireev I., Evfratov S., Rubtsova M., Belova E., Strelkova O., Cherepaninets V., Zhironkina O. (2017). Interstitial telomeric repeats-associated DNA breaks. Nucleus.

[52] Rovatsos M.T., Marchal J.A., Romero-Fernández I., Fernandez F.J., Giagia-Athanosopoulou E.B., Sánchez A. (2011). Rapid, independent, and extensive amplification of telomeric repeats in pericentromeric regions in karyotypes of arvicoline rodents. Chromosome Res..

[53] Augstenová B., Mazzoleni S., Kratochvíl L., Rovatsos M. (2017). Evolutionary dynamics of the W chromosome in caenophidian snakes. Genes.

[54] Rovatsos M., Marchal J., Giagia-Athanasopoulou E., Sánchez A. (2021). Molecular composition of heterochromatin and its contribution to chromosome variation in the *Microtus thomasi*/*Microtus atticus* species complex. Genes.

[55] Sumner A.T. (1972). A simple technique for demonstrating centromeric heterochromatin. Exp. Cell Res..

[56] Endow S. (1982). Polytenization of the ribosomal genes on the X and Y chromosomes of *Drosophila melanogaster*. Genetics.

[57] Ijdo J.W., Baldini A., Ward D.C., Reeders S.T., Wells R.A. (1991). Origin of human chromosome 2: An ancestral telomere-telomere fusion. Proc. Natl. Acad. Sci. USA.

